
JOURNAL INFORMATION
==============================
NLM Title Abbreviation: Gastroenterol Rep (Oxf)
Journal ID: Gastroenterol Rep (Oxf)
NLM Journal ID: 101620508
Journal ID: gastro

Gastroenterology Report

EISSN: 2052-0034
Publisher: Oxford University Press

ARTICLE INFORMATION
==============================
PMCID: PMC4760070
DOI: 10.1093/gastro/gow001
Article ID: gow001
Article version: 1
Subjects: Chinese Abstracts

Chinese Abstracts – Volume 4 Issue 1*

*This article abstract was originally published in English. This translation has not been verified and should not be relied upon—it is provided for reference purposes only. The Publishers have not checked this translation and accept no liability for completeness or accuracy of this translation or the use of this translation for whatever purpose. This translation may be incomplete and inaccurate in whole or in part. If you need to rely upon a translation of this abstract, a professional human translator should be engaged to supply an accurate translation of the original English. When referencing articles from this journal, please always refer to the original English version, rather than a translated equivalent.

Print publication date: 2016 Feb
Electronic publication date: 2016 Feb 18
Volume: 4
Issue: 1
First page: C1
Last page: C3
Copyright: © The Author(s) 2016. Published by Oxford University Press and the Digestive Science Publishing Co. Limited.
Copyright year: 2016
License: This is an Open Access article distributed under the terms of the Creative Commons Attribution License (http://creativecommons.org/licenses/by/4.0/), which permits unrestricted reuse, distribution, and reproduction in any medium, provided the original work is properly cited.
License URL: https://creativecommons.org/licenses/by/4.0/

Page count: 3

==============================
OXFORD

*Gastroenterology Report*, 2016, 1–3

doi: 10.1093/gastro/gow001

### Chinese Abstracts – Volume 4 Issue 1*

### Review（综述）

### Extra-hepatic manifestations associated with hepatitis E virus infection: a comprehensive review of the literature
### 戊型肝炎病毒感染的肝外表现：文献综述

[摘要] 背景与目的：戊型肝炎病毒（HEV）感染是威胁公众健康的一个重要问题，每年受感染人数约 2 千万，其中 250 万人出现急性肝损伤，56000 人死亡。与其他病毒性肝炎一样，肝外表现是该病的一个重要特征。急性腺胰炎、神经系统、肌肉骨骼系统、血液系统、肾脏及其他免疫调节疾病作为 HEV 肝外表现已有过报道，而且仍然不时有新的肝外表现被发现。本文旨在全面综述 HEV 感染肝外表现的相关文献。

数据收集：以 MeSH 主题词“hepatitis E ”和每一项 HEV 相关的肝外表现作为关键词，在 PubMed 数据库中进行检索，不设置任何语种或资料限制。并通过纳入文献的文后参考文献列表追加检索。

结果：既往文献曾报道过多种与 HEV 感染相关的肝外表现，其中一些肝外表现与HEV感染之间存在相关性，而且排除了这些肝外表现的其他可能病因，提示 HEV 可引起这些肝外表现。根据现有的数据， HEV 感染似乎与急性胰腺炎、神经系统疾病（原发性外周支配神经受累，最常见的是继发于神经痛性肌萎缩的 Guillain-Barre 综合征）、血液系统疾病（磷酸葡萄糖脱氢酶缺乏引起的溶血性贫血和严重的血小板减少症）、肾小球肾炎及混合型冷球蛋白血症等疾病密切相关。而 HEV 感染是否与肌肉骨骼系统或其他免疫调节疾病具有相关性尚需更多数据证实。

结论：对于急性胰腺炎、 Guillain-Barre 综合征、神经痛性肌萎缩、磷酸葡萄糖脱氢酶缺乏引起的溶血性贫血、严重血小板减少症、肾小球肾炎或混合型冷球蛋白血症患者，应考虑 HEV 感染的可能。同时，对于急性或慢性 HEV 感染者，也应留意上述肝外疾病的症状和体征。而对于 HEV 在其他肝外疾病中的作用尚需更多数据证实。

[关键词] 戊型肝炎病毒；病毒性肝炎；肝外表现；疫苗；一级预防

### Imaging lexicon for acute pancreatitis: 2012 Atlanta Classification revisited
### 急性胰腺炎的影像学术语：基于 2012 亚特兰大分类标准的解读

[摘要] 2012年，在多个国家及国际学会的支持下，亚特兰大工作组基于网络共识对急性胰腺炎 Atlanta 分类系统（1992 年版）进行了修订。修订版明确了急性胰腺炎的两个病期：早期和晚期。急性胰腺炎可分为间质水肿性胰腺炎和坏死性胰腺炎两类。其严重程度可依照器官衰竭和局部/系统并发症情况分为轻度、中重度和重度三个水平。根据胰腺炎的类型，胰周积聚可分为急性胰周积液、胰腺假性囊肿和包裹性坏死。熟悉修订版中的术语对于影像学结果的准确交流是十分必要的。本文将对这些更新的术语进行总结，并通过图例来呈现相应的影像学结果。

[关键词] 急性胰腺炎；影像学；急性胰周积液；假性囊肿；包裹性坏死

### Steatosis and hepatitis C
### 脂肪变性与丙型肝炎

[摘要] 丙型肝炎病毒（HCV）感染是一种全球范围内的常见肝病，大多数（75%－80%）的感染者将转为慢性。 HCV 的慢性特征常导致脂肪肝、肝硬化和肝细胞癌。由于病毒因素（病毒蛋白对某些细胞间通路的影响）和宿主因素（体重过重、胰岛素抵抗、糖尿病和酗酒）的共同作用，多数 HCV 患者（55%）出现脂肪肝。基因 1 型 HCV （美国最常见的 HCV 基因类型）对聚乙二醇干扰素（PEG-IFN）和病毒唑（RBV）的治疗反应率较低，长期治疗反应率不足 40%。将直接抗病毒药物（新近通过 FDA 批准上市）加入标准治疗方案中可以提高治疗反应率。然而， HCV 相关的终末期肝病仍然是美国肝移植的首要适应证。本文重点探讨 HCV/脂肪肝与代谢综合征的相关性。

[关键词] 丙型肝炎病毒；脂肪肝；代谢综合征

*This article abstract was originally published in English. This translation has not been verified and should not be relied upon—it is provided for reference purposes only. The Publishers have not checked this translation and accept no liability for completeness or accuracy of this translation or the use of this translation for whatever purpose. This translation may be incomplete and inaccurate in whole or in part. If you need to rely upon a translation of this abstract, a professional human translator should be engaged to supply an accurate translation of the original English. When referencing articles from this journal, please always refer to the original English version, rather than a translated equivalent.
本文摘要最初是以英文发表。中文摘译未获认证，谨供参考。出版社没有对中文摘译进行核验，不负责译文的完整性和准确性，也不会将其用以其他目的。中文摘译可能会出现部分不完整或不准确的情况。如果你需要准确获悉摘要，最好能请一位专业译者针对英文原文进行翻译。阅读杂志文章，请参考英文原文，不推荐中文翻译。

### Impact of anaesthetic technique on survival in colon cancer: a review of the literature
### 麻醉技术对结肠癌患者术后生存期的影响：文献综述

[摘要] 对于潜在可根治的结肠癌，手术切除是核心治疗方式。尽管肉眼看不见，但大多数患者术中都会残留一些癌细胞。而手术本身所引起的免疫抑制也会影响到肿瘤复发和术后生存。相较于阿片类麻醉剂，区域阻滞麻醉或椎管内麻醉（如硬膜外麻醉）或可通过减少应激和降低阿片类麻醉剂的用量，减轻对免疫功能的抑制作用。研究显示，区域阻滞麻醉可降低乳腺癌和前列腺癌的复发率，从而印证了这一假设。然而对于结肠癌，相关研究结果却不尽一致。本文对所有探讨硬膜外麻醉与结肠癌手术患者生存相关性的研究进行了综述。

[关键词] 结肠癌；硬膜外麻醉；免疫抑制；生存

### Original article（论著）

### The 2 + 1 paradigm: an efficient algorithm for central reading of Mayo endoscopic subscores in global multicenter phase 3 ulcerative colitis clinical trials
### “2+1”模式：一项有效评估溃疡性结肠炎全球多中心三期临床试验中Mayo内镜评分的中心读取流程

[摘要] 尽管中心读取（central reading）在临床试验中非常重要且对研究结果具有潜在影响，但其在炎性肠病（IBD）临床试验的相关文献中仍然重视不够。尽管目前有些 IBD 研究中采用了中心读取，但在方法学上对于如何读取并未作详细交待。我们在此提出了几项关键原则，用以设计出一项行之有效的应用于溃疡性结肠炎（UC）研究的中心读取模式，这一模式无论在研究管理、实际操作或是临床实践中都能有效应用。为了制定一项标准化的读取方法以满足日益增多的多中心三期 IBD 临床试验，我们遵照这些原则设计出了一项优化的中心读取方法，称之为“2+1”模式。该模式涉及各分站及中心站读取者、有效的评分标准、多项盲法读取措施，所有这些都有助于减少偏倚和获得可靠的内镜评分以反映疾病严重程度。由于具有很强的可操作性，该模式可在临床试验中作为一项标准操作流程。该模式应用于临床试验可为内镜评估提供合理的方法，应引起研究赞助方、合同研究组织、主要研究者及监督机构足够的重视，应在临床研究中常规应用并进一步评估。

[关键词] 溃疡性结肠炎；内镜；临床试验

### Redo sphincteroplasty: are the results sustainable?
### 再次括约肌成形术：效果能长久吗？

[摘要] 目的：本研究旨在调查再次括约肌成形术（RS）患者的长期结局。

方法：利用一个前瞻性数据库对 1988 年 11 月至 2011 年 12 月间接受 RS 手术的排便失禁患者进行回顾性分析。通过问卷和电话调查进行克利夫兰临床排便失禁评分（CCFFIS，0分最佳，20 分最差）和排便失禁生活质量评分（FIQoL，4.1 分最佳，1 分最差）。手术成功定义为截至末次随访未进一步行排便手术、无造口且 CCFFIS 评分 <9。计量资料的比较采用 Wilxoxon 和 Mann-Whitney U 检验。采用双元逻辑回归分析来确定手术成功的预测因素。

结果： 84 例患者接受 RS 手术，其中 56 例（66.7%）获得 74 月的中位随访（12 – 283 月）。末次随访时，平均 CCFFIS 评分由 16.5 ± 3.7 下降至 11.9 ± 6.6 （$P < 0.001$）。12 例（21.4%）患者接受后续便秘手术，3 例（5.4%）进行了永久性造口，判定为手术失败。随访结束时，18 例（32.1%）患者 CCFFIS 评分 <9，16 例获得长期成功。24 例患者接受了 FIQoL 评分，平均2.6分（1.04.1分）。术后CCFFIS评分与FIQoL显著负相关（Spearman 相关系数为–0.854, $P < 0.001$）。逻辑回归分析并没有发现任何预测 RS 成功的临床参数。

结论：基于我们的成功标准，RS 手术的长期成功率（中位随访时间 74 月）并不理想。

[关键词] 排便失禁；再次括约肌成形术；长期结局

### Comparison of outcomes for patients with primary sclerosing cholangitis associated with ulcerative colitis and Crohn’s disease
### 合并溃疡性结肠炎与合并克罗恩病的原发性硬化性胆管炎患者临床结局比较

[摘要] 背景：合并原发性硬化性胆管炎（PSC）的溃疡性结肠炎（UC）与克罗恩病（CD）的结局比较尚不清楚。本研究旨在对两者的临床结局进行客观比较。

方法：273例合并PSC的炎性肠病患者（UC 223 例， CD 50 例）纳入研究。收集入组病例的临床及人口统计学资料。

结果：两组病例 PSC 危险度评分相近。 PSC-UC 组和 PSC-CD 组患者的中位随访时间分别是 12（0 – 38）年和14 （1 – 36）年。中位疾病复发次数 PSC-UC 组多于PSC-CD组[1 （0 – 20） *vs.* 0 （0 – 9）; $P < 0.001$]。出现结肠瘤变的患者比例 PSC-UC 组高于 PSC-CD 组（35.9% *vs.* 18%; $P = 0.009$）。结肠切除手术的比例风险模型分析显示， PSC-UC 患者结肠切除手术风险较 PSC-CD 增高 12% [风险比 (HR) = 0.88; 95% 可信区间 (CI) 0.51 – 1.51; $P = 0.64$] 。同时，针对 PSC 的肝移植可降低结肠切除手术风险（HR = 0.57; 95% CI 0.37 – 0.89; $P = 0.013$）；而结肠瘤变则增加该风险（HR = 3.83; 95% CI 2.63 – 5.58; $P < 0.001$）。结肠瘤变的比例风险模型显示， PSC-UC 患者结肠瘤变风险较 PSC-CD 患者增高 56% （HR = 0.44; 95% CI 0.16 – 1.25; $P = 0.12$）。

结论：合并 CD 的 PSC 患者结肠瘤变和结肠切除手术的风险低于合并 UC 者。

[关键词] 原发性硬化性胆管炎；溃疡性结肠炎；克罗恩病；结肠瘤变；结肠切除术；肝移植

### Comparison of abdominal ultrasound, endoscopic ultrasound and magnetic resonance imaging in detection of necrotic debris in walled-off pancreatic necrosis
### 腹部超声、内镜超声与磁共振成像对于包裹性胰腺坏死碎片诊断价值的比较

[摘要] 背景：包裹性胰腺坏死（WOPN）是急性胰腺炎一个重要并发症，主要是通过内镜超声（EUS）或磁共振成像（MRI）等影像学检查来诊断，这些检查可清晰见到坏死碎片。而腹部超声（USG）对于这些坏死碎片的发现价值目前尚不清楚。

方法： 52 例 WOPN 患者（其中男性 37 例，年龄38.9 ± 12.6岁）被前瞻性纳入研究，分别接受 EUS 、 MRI 和USG检查。这些检查于急性胰腺炎发病后11.7 ± 5.5周进行，并于2天内完成。

结果：所有患者均可通过 EUS 和 MRI 诊断 WOPN ，而有4例（7.6%）通过 USG 未能发现 WOPN （其中3例是固体碎片为主的坏死灶，另1例坏死灶中存在空气）。在所有发现的WOPN中，三项检查均能检测到固体坏死碎片。EUS 和 USG 在10例（20%）患者中检测到< 10%的固体碎片，而 MRI 这一检测比例为27%（14例）。EUS 和USG在33例（63%）患者中检测到的固体碎片在10% – 40%之间， MRI 的这一检测比例为58%（30例）。当固体固体碎片比例 > 40%时，EUS 和 MRI 则更具优势，其在8例（15%）患者中检测到 > 40%的固体碎片，而 USG 这一检测比例为11%（5例）。EUS还可以发现坏死周围组织，而 USG 和 MRI 却不能。

结论： USG 能对大多数 WOPN 进行诊断，其准确率与EUS和MRI相当。然而， ESG 的局限性在于检测WOPN周围组织及诊断固体碎片比例较高或内含空气的 WOPN 的能力不足。

[关键词] 内镜超声；腹部超声；磁共振成像；胰腺坏死；急性胰腺炎

### Histopathological study using computer database of 10 000 consecutive gastric specimens: (2) malignant lesions

### 电脑数据库中10,000例连续胃组织标本的组织病理学研究：（2）恶性病变

[摘要]　通过电脑数据库，作者对一家日本大型医院病理实验室2002－2013年间的10,000例连续胃组织标本进行组织病理学分析。所有组织切片被回顾性观察，这10,000例胃组织标本中良性病变8579例，恶性病变1421例。恶性病变中胃癌1342例（94.4%），胃肠间质瘤（GIST）34例（2.4%），黏膜相关淋巴组织（MALT）淋巴瘤25例（1.8%），非霍奇金恶性淋巴瘤19例（1.3%），转移癌1例（0.1%）。在1342例胃癌中，管状腺癌755例，乳头状腺癌176例，黏液腺癌147例，印戒细胞癌145例，低/未分化腺癌114例，腺鳞癌4例，转移性肺小细胞癌1例。在手术切除标本中，早期胃癌258例，进展期胃癌521例。在34例 GIST 中，2例为上皮细胞型，32例为梭形细胞型； GIST 大小为1－15 cm ，平均5.6 cm； KIT （CD117）表达均为阳性， CD34 阳性31例，desmin阳性2例，S100阳性4例。6例GIST进行了基因检测，均显示KIT和/或PDGFRA 点突变。在25例 MALT 淋巴瘤中均可见到中心细胞样细胞和淋巴上皮病变；92%的病变可发现幽门螺杆菌感染。在19例非霍奇金淋巴瘤中，17例为弥漫性大B细胞淋巴瘤，1例为外周T细胞淋巴瘤，1例为NK细胞淋巴瘤。

[关键词] 胃；恶性病变；细胞病理学

### Genome-wide gene expression analysis for target genes to differentiate patients with intestinal tuberculosis and Crohn's disease and discriminative value of FOXP3 mRNA expression

### 肠结核与克罗恩病差异表达基因的全基因分析及FOXP3 mRNA 表达的鉴别价值

[摘要] 背景：克罗恩病（CD）和肠结核（ITB）是有着相似表型的慢性肉芽肿性疾病，因此，找到一种生物标志物来鉴别这两种疾病是必要的。本研究旨在对 ITB 和 CD 的结肠标本进行全基因表达分析。同时，为了评价调节性 T 细胞的作用，我们对血清标本及结肠标本进行FOXP3 mRNA 定量检测。

方法：收集33名研究对象（CD 患者、 ITB 患者及正常对照）血清和结肠组织配对样本，提取标本中的总RNA。采用 Illumina HumanWG-6 BeadChip 试剂盒对3组研究对象中的6种总RNA样本进行人类全基因组表达芯片分析。采用 real-time PCR 对具有合格 RNA 样本的病例（CD 4例，ITB 5例，正常对照4例）血清和组织标本进行 FOXP3 mRNA 表达水平检测。

结果：在 CD 和 ITB 患者中，分别有92和382个基因表达上调1.5倍，91和256个基因表达下调1.5倍。过氧化物酶体增殖物介导的PPARγ 信号通路在 CD 中下调最为显著($P < 0.005$)；此外， IL4/5/6 信号通路和 Toll 样受体信号通路也存在显著差异表达，上调2倍以上。而在 ITB 患者上调最为显著是补体活化通路尤其是经典通路。ITB患者结肠组织标本中FOXP3 mRNA表达水平明显高于CD患者（4.70 ± 2.21 *vs* 1.48 ± 0.31, $P = 0.016$）。

结论：结肠黏膜FOXP3 mRNA表达可作为鉴别 ITB 与 CD 的一项标志物。 ITB 患者补体激活通路的上调提示 ITB 的发病机制与肺结核相似。 CD 患者中 PPARγ 通路的下调提示 PPARγ 依赖的微生物屏障功能可能成为新的治疗靶点。

[关键词]　克罗恩病；肠结核；基因表达芯片；信号通路；FOXP3 mRNA

### Case report（病例报告）

### Portal biliopathy, magnetic resonance imaging and magnetic resonance cholangiopancreatography findings: a case series

### 门脉性胆道病、磁共振成像和磁共振胆胰管造影：病例系列报告

### Auto-inflammatory diseases in ileal pouch patients with NOD2/CARD15 mutations

### NOD2/CARD15 突变的回肠储袋患者的自身免疫性疾病

### *Cutis laxa* presenting as recurrent ileus

### 皮肤松弛症表现为复发性肠梗阻

### Small bowel malignant melanoma presenting as a perforated jejunal diverticulum: a case report and literature review

### 小肠恶性黑色素瘤表现为空肠憩室穿孔：病例报告及文献复习

### A rare cause of gastro-intestinal hemorrhage in a patient with a Roux-en-Y gastric bypass

### Roux-en-Y胃旁路术患者胃肠出血的一个罕见原因
